# Dietary Patterns and Frailty in Older Korean Adults: Results from the Korean Frailty and Aging Cohort Study

**DOI:** 10.3390/nu13020601

**Published:** 2021-02-12

**Authors:** Jinhee Kim, Yunhwan Lee, Chang Won Won, Mi Kyung Kim, Seunghee Kye, Jee-Seon Shim, Seungkook Ki, Ji-hye Yun

**Affiliations:** 1 Department of Preventive Medicine and Public Health, Ajou University School of Medicine, 164 World cup-ro, Youngtong-gu, Suwon 16499, Korea; jhkim06@ajou.ac.kr (J.K.); donquixote@outlook.com (S.K.); dream10307@naver.com (J.-h.Y.); 2 Institute on Aging, Ajou University Medical Center, 164 World cup-ro, Youngtong-gu, Suwon 16499, Korea; 3 Elderly Frailty Research Center, Department of Family Medicine, College of Medicine, Kyung Hee University, 23 Kyung Hee Dae-ro, Dongdaemun-gu, Seoul 02447, Korea; chunwon@khmc.or.kr; 4 Department of Preventive Medicine, College of Medicine, Hanyang University, 222 Wangsimni-ro, Seongdong-gu, Seoul 04763, Korea; kmkkim@hanyang.ac.kr; 5 Nutrition Education, Graduate School of Education, Gachon University, 1342 Seongnamdae-ro, Sujeong-gu, Seongnam 13120, Korea; shkye2@gmail.com; 6 Cardiovascular and Metabolic Diseases Etiology Research Center, Yonsei University College of Medicine, 50-1 Yonsei-ro, Seodaemun-gu, Seoul 03722, Korea; SHIMJS@yuhs.ac; 7 Department of Preventive Medicine, Yonsei University College of Medicine, 50-1 Yonsei-ro, Seodaemun-gu, Seoul 03722, Korea

**Keywords:** dietary patterns, reduced rank regression, frailty, community-dwelling older people

## Abstract

There are few studies on dietary patterns and frailty in Asians, and the results are controversial. Therefore, this study examined the association between dietary patterns and frailty in older Korean adults using the Korean Frailty and Aging Cohort Study (KFACS). The sample consisted of 511 subjects, aged 70–84 years, community-dwelling older people from the KFACS. Dietary data were obtained from the baseline study (2016–2017) using two nonconsecutive 24-h dietary recalls, and dietary patterns were extracted using reduced rank regression. Frailty was measured by a modified version of the Fried Frailty Phenotype (FFP) in both the baseline (2016) and the first follow-up study (2018). A logistic regression analysis was used to examine the association between dietary patterns and frailty status in 2018. The “meat, fish, and vegetables” pattern was inversely associated with pre-frailty (OR = 0.41, 95% CI = 0.21–0.81, *p* for trend = 0.009) and exhaustion (OR = 0.41, 95% CI = 0.20–0.85, *p* for trend = 0.020). The “milk” pattern was not significantly associated with frailty status or the FFP components. In conclusion, a dietary pattern with a high consumption of meat, fish, and vegetables was associated with a lower likelihood of pre-frailty.

## 1. Introduction

Frailty is a condition whereby decreased homeostatic reserves result in adverse reactions to stress, as a result of age-related decline in many physiological systems [1]. In older adults, frailty leads to poor health outcomes such as impaired cognitive function, falls, fracture, physical disability, hospitalization, and mortality [1]. With rising healthcare costs and increases in the life expectancy of older people with frailty [2], prevention of frailty is crucial for successful aging.

Among the modifiable risk factors associated with frailty, the role of diet has been examined for frailty prevention [3]. Previous studies have mainly focused on specific single nutrients or foods, and they reported that vegetables, fruits, whole grains, and low-fat dairy products are inversely related to frailty [3]. However, people eat meals that contain a combination of nutrients and a variety of foods. Nutrients and foods create interactions within the body that can affect health outcomes; therefore, identifying dietary patterns may be more beneficial for preventing frailty [4]. In Western countries, a recent meta-analysis showed that the Mediterranean diet, a priori-defined dietary pattern, is associated with reduced incidence of frailty in older people [5]. Various a posteriori dietary patterns [4] have been derived using principal component analysis, factor analysis, or cluster analysis, and their association with frailty has been investigated [6,7,8]; however, previous results from studies on diets in Western countries are different from those relating to the Asian diet. Moreover, although a few studies [9,10,11] have examined the relationship between dietary patterns and frailty in Asian countries, the results are controversial.

Therefore, we examined the association between dietary patterns extracted using reduced rank regression (RRR) and frailty in older Korean community-dwelling residents. RRR reduces the dimensions of the predictor variables and maximizes the variation of the response variables, reflecting both a priori-defined and posteriori-derived dietary patterns [12].

## 2. Materials and Methods

### 2.1. Study Population

Data were retrieved from the Korean Frailty and Aging Cohort study [13]. The subjects were enrolled according to age- and gender-specific strata from South Korean, community-dwelling older people, aged 70–84 years. Baseline surveys were conducted from May to November 2016 across eight university-affiliated hospitals and two public health centers (n = 1559). Dietary intake was examined during home visits on two nonconsecutive days for a sub-cohort study including two-thirds of the baseline subjects from September 2016 to November 2017. The first follow-up surveys were conducted from March to December 2018. The study was performed following the tenets of the Helsinki Declaration, and it was approved by the Institutional Review Board of Ajou University Hospital (AJIRB-SBR-SUR-20-356). Written informed consent was obtained from all subjects. Of the 580 who completed all evaluations, those with missing values (n = 69) for educational level, marital status, smoking, number of physician-diagnosed chronic diseases, depression index, and cognitive function were excluded. The final analytical sample included 511 subjects.

### 2.2. Assessment of Dietary Intake

Dietary data were obtained from the baseline surveys (2016–2017) using two nonconsecutive 24-h dietary recalls, which were carried out by trained interviewers during home visits over 3–10 month intervals. Bowls, plates, and food pictures, developed by the National Institutes of Health (NIH) and the Korea Disease Control and Prevention Agency (KDCA), were used to estimate portion size. Trained interviewers examined the names and amount of food consumed, the types of meals, and eating locations of the previous day. Food and nutrient intakes were calculated using the 24-h recall dietary assessment system of the NIH and KDCA based on the National Rural Living Science Institute database [14]. Individual foods were grouped into 22 food groups based on similar nutritional content and characteristics [14].

### 2.3. Assessment of Frailty

Frailty status was measured by a modified version of the Fried Frailty Phenotype (FFP) [15] in both the baseline (2016) and the first follow-up survey (2018). It contains five components: weight loss (unintentional, 4.5 kg or more in the previous year), self-reported exhaustion (felt that everything was an effort or that one could not get going ≥ 3 times a week), low physical activity, measured by the International Physical Activity Questionnaire Short Form (Korean version) [16] (<494.65 kcal/week for men, < 283.50 kcal/week for women) [17], low grip strength (Takei dynamometer, with body mass index (BMI) < 22.0 then ≤ 25.4 kg, 22.0 ≤ BMI ≤ 23.9 then ≤ 27.1 kg, 24.0 ≤ BMI ≤ 25.9 then ≤ 27.8 kg, BMI ≥ 26.0 then ≤ 28.5 kg for men, and BMI < 23.0 then ≤ 16.8 kg, 23.0 ≤ BMI ≤ 24.9 then ≤ 17.6 kg, 25.0 ≤ BMI ≤ 26.9 then ≤ 17.8 kg, BMI ≥ 27.0 then ≤ 17.7 kg for women) [13], and slow gait speed (if height ≤ 165.0 cm then ≤ 0.93 m/sec and if height > 165.0 cm then ≤ 0.98 m/sec for men, and if height ≤ 152.0 cm then ≤ 0.85 m/sec and if height > 152.0 cm then ≤ 0.93 m/sec for women) [13]. Each component was assigned a score of 1 (if present) or 0 (if absent). Frailty scores ranged from 0 to 5, and frailty status was categorized as robust (0), pre-frail (1–2), and frail (3–5). We used the frailty incidence data (excluding the subjects classified as frail in 2016), the remaining subjects’ frailty status in 2018 was measured, and these were included in the analysis.

### 2.4. Covariates

Baseline data (2016) were used for covariates. Age, gender, education, and marital status were included for demographic characteristics. Body mass index was calculated as kg/m^2^. Physician-diagnosed chronic diseases included imbalances of the circulatory system, musculoskeletal system and its connective tissue, respiratory, digestive, endocrine, nervous, and urogenital systems as well as neoplasms, and diseases were classified as 0, 1, and ≥2. The number of prescription drug treatments was categorized as < 4 and ≥ 4. The Korean version of the Short Form Geriatric Depression Scale (SGDS-K) [18] was used as a depression index (depression ≥ 8 points), and the Mini-Mental State Examination in the Korean version of the CERAD Assessment Packet (MMSE-KC) [19] was used as a cognitive function index (normal ≥ 24 points). Falls (experience of falling over in the past year) were also included. Smoking, dietary supplement use, and energy intake were included for health behaviors.

### 2.5. Statistical Analysis

The dietary patterns were extracted using RRR, and the RRR method is described elsewhere [12]. Briefly, the purpose of this approach is to reduce the dimension of the predictor variables (food groups) and maximize the variation of the response variables (usually nutrients or biomarkers) that are hypothesized to be associated with the outcome. Intake of protein and vitamin D have been associated with frailty in a previous study [3] and these were selected as responses for RRR. Two dietary patterns were extracted using RRR from 22 food groups. The relationship between the food groups and the dietary patterns was designated by factor loadings. Food groups with factor loadings ≥0.2 were considered positive contributors to the patterns, and foods with factor loadings up to −0.2 were negative contributors to the patterns. Dietary pattern scores were classified into tertiles.

The relationships between the dietary pattern and subject characteristics or between frailty status and subject characteristics were analyzed using the analysis of variance and post-hoc tests, chi-square tests and were presented as the mean ± standard deviation or a number (percentage). After adjusting for covariates, a multinomial logistic regression analysis was used for the association between dietary patterns and frailty, and a logistic regression analysis was used for the association between dietary patterns and the individual FFP. *p*-values for trend were also estimated according to the dietary pattern tertiles. The odds ratio (OR) and 95% confidence interval (CI) were calculated. We also added the results of the analysis using a continuous variable (dietary pattern scores divided by standard deviation). The *p*-values reported were two-sided, and the significance level was set at <0.05. All statistical analyses were conducted using SAS version 9.4 (SAS Institute, Inc., Cary, NC, USA).

## 3. Results

Two dietary patterns were obtained using RRR with factor loadings for each food group (Table 1). The first dietary pattern, labeled the “meat, fish, and vegetables” pattern, was characterized by high consumption of meat, fish, vegetables, rice, poultry, oils and fats, sugars and sweets, and seasonings. The second pattern, labeled as the “milk” pattern, was characterized by high consumption of milk and dairy products, fish, eggs, and nuts and seeds, and low consumption of noodles and dumplings, meat, poultry, beans, and seasonings. The two dietary patterns combined explained 13.7% of the total variation in the food group consumption and 64.2% of the variation in the response variables (protein and vitamin D).

The study subjects had an average age of 75.9 ± 4.0 years, and 50.5% were women. Of the subjects, 43.1%, 50.7%, and 6.3% were robust, pre-frail, and frail, respectively, in 2018 (data not shown). The relationship between the dietary pattern score and subject characteristics is shown in Table 2. For the “meat, fish, and vegetables” pattern, subjects in the highest tertile of the dietary pattern score were more likely men, highly educated, married, and they were less likely to have physician-diagnosed chronic diseases. They also had lower depression scores (SGDS-K ≥ 8 points), higher normal cognitive function (MMSE-KC ≥ 24 points), and had higher energy intake than those in the lowest tertile. For the “milk” pattern, gender, education, dietary supplement use, and energy intake were significantly different among the tertiles of dietary pattern scores.

The relationship between frailty status and subject characteristics is shown in Table 3. Compared with those who were robust, frail subjects tended to be older, women, less educated, not married, with more physician-diagnosed chronic diseases, and had received more prescription drug treatments. They also had higher depression scores (SGDS-K ≥ 8 points), lower normal cognitive function (MMSE-KC ≥ 24 points), and had less energy intake.

Table 4 shows the association between the dietary pattern score and frailty. In the “meat, fish, and vegetables” pattern, subjects in the highest tertile of the dietary pattern score were less likely to have pre-frailty compared to those subjects in the lowest tertile, after adjusting for covariates (OR = 0.41, 95% CI = 0.21–0.81, *p* for trend = 0.009). Subjects in the highest tertile of the dietary pattern score were less likely to have frailty compared to those subjects in the lowest tertile (unadjusted) (OR = 0.14, 95% CI = 0.04–0.43, *p* for trend <0.001), but the significant association disappeared after adjusting for covariates. In regard to the individual components of the FFP, the increasing tertiles of the dietary pattern score were inversely associated with exhaustion (OR = 0.41, 95% CI = 0.20–0.85, *p* for trend = 0.020). The “milk” pattern was not significantly associated with either the frailty status or the individual components of the FFP criteria. In addition, the result of the relationship between the continuous change in the dietary pattern scores (dietary pattern scores divided by the standard deviation) and frailty status was similar to the above.

## 4. Discussion

In the current study, two dietary patterns were extracted using RRR. The “meat, fish, and vegetables” pattern was inversely associated with pre-frailty and exhaustion after adjustment for covariates. The “milk” pattern was not significantly associated with either the frailty status or the individual components of the FFP criteria.

To our knowledge, only three previous studies have investigated the association between dietary patterns and frailty in Asian populations [9,10,11]. A Chinese prospective study [9] showed that a “vegetable-fruit” dietary pattern was not related to frailty incidence. A Taiwanese cross-sectional study [10] reported that the RRR-derived dietary pattern, which included a high consumption of fruit, nuts and seeds, tea, vegetables, whole grains, shellfish, milk, and fish, was inversely associated with frailty. A Japanese prospective cohort study [11] showed that a “protein-rich” dietary pattern was negatively related to frailty while a “salt and pickles” pattern and “sugar and fat” pattern were positively related to frailty. In Western countries, most studies have examined the association between the Mediterranean diet (a priori-defined dietary pattern) and frailty. A recent meta-analysis showed that the Mediterranean diet was associated with lower frailty incidence [5]. While few studies have examined the association between a posteriori-derived dietary patterns and frailty, in a Spanish prospective study [6], “prudent” dietary patterns (high intake of olive oil and vegetables) were inversely associated with frailty incidence. In the Three-City Bordeaux Study [7], men in the “pasta” pattern and women in the “biscuits and snacking” pattern had higher rates of frailty than those in the “healthy” pattern (higher fish intake in men and higher fruits and vegetables intake in women). In the longitudinal results of the Rotterdam study [8], adherence to the “traditional” pattern (high in legumes, eggs and savory snacks) was associated with less frailty. The results of this current study are partially similar to those of the previous studies.

Several potential mechanisms related to dietary pattern and frailty have been suggested. Studies of nutrient intake and frailty have mainly focused on proteins, which stimulate muscle protein synthesis. The results have reported an inverse relationship between protein intake and frailty in older people [20,21]. In our study, higher consumption of protein-rich foods, such as meat, fish, and poultry were associated with reduced pre-frailty. Vitamin D is involved in frailty through two metabolic pathways, bone mineralization and muscle strength [3,22]. The antioxidant properties may delay the development of frailty by preventing oxidative stress [23]. Many studies have reported inverse associations between frailty and micro-nutrients such as carotenoids, vitamin C, vitamin E, and selenium [24,25]. Therefore, vegetables may contain antioxidative properties related to frailty prevention. The current study indicated that higher consumption of seasonings (mainly soy sauce, red pepper powder, Gochujang [fermented red pepper paste], Doenjang [soybean paste], table salt) was associated with lower pre-frailty. Although, we could not determine the mechanism by which these dietary factors reduce pre-frailty, the high consumption of seasonings involved a more balanced macronutrient composition than low consumption of seasonings [26], and this may have beneficial effect in preventing pre-frailty (Table S1).

The “meat, fish, and vegetables” pattern was not significantly associated with frailty after adjusting for covariates, which may be because of the low number of frail subjects (n = 32). A previous study reported that the consumption of dairy products may decrease frailty (mean intake of total dairy products: 306.3 g per day) [27]. However, in the current study, the “milk” pattern was not associated with frailty. The consumption of milk and dairy products was low (mean intake: 52.9 g per day, data not shown) and the low value of the explained variation for the “milk” pattern could partly explain the nonsignificant association with frailty status and FFP criteria.

This study has several potential limitations. First, dietary data were obtained using 24-h dietary recalls. This method cannot accurately reflect the usual dietary intake of study subjects. However, we examined the dietary intake for two nonconsecutive days and used the mean intake. Second, dietary patterns are highly related to the study population’s diet. Therefore, the dietary patterns extracted from this study may not be generalizable to other populations, especially those of different cultures. Third, participants with cognitive impairment may be limited for 24-h dietary recalls and their estimation of intake may not be reliable. Fourth, although we included as many potential confounders as possible, residual confounding may remain. Lastly, although the current study focused on the relationship between physical frailty and dietary patterns, there are multidimensions of frailty and multidomain relevance of healthy behaviors related to frailty, so these should be considered in the future [28,29].

## 5. Conclusions

The “meat, fish, and vegetables” pattern was significantly associated with lower odds of pre-frailty. Our results suggest a potentially protective effect of a “protein-rich, vegetables” dietary pattern against frailty.

## Figures and Tables

**Table 1:** Factor loading values derived by reduced rank regression ^a^ (*n* = 511).

Food Groups	Meat, Fish, and Vegetables Pattern	Milk Pattern
Rice	**0.24**	−0.16
Rice cakes	−0.04	−0.08
Other grains	0.01	−0.12
Noodles & dumplings	0.10	−**0.24**
Flour & bread	0.12	0.07
Potatoes & starches	0.08	−0.08
Meat	**0.35**	−**0.36**
Poultry	**0.22**	−**0.24**
Fish	**0.41**	**0.30**
Eggs	0.17	**0.26**
Beans	0.17	−**0.20**
Shellfish	0.10	0.07
Nuts & seeds	0.18	**0.23**
Vegetables	**0.37**	−0.09
Kimchi	0.18	−0.18
Fruits	0.07	−0.01
Milk & dairy products	0.13	**0.55**
Oils & fats	**0.21**	0.01
Sugars & sweets	**0.25**	0.02
Beverages	0.10	−0.01
Alcohol	0.06	−0.19
Seasonings	**0.41**	−**0.23**
Explained variation (%)		
Food groups	8.3	5.4
Responses	51.2	13.0

^a^ Factor loadings with absolute values ≥ 0.20 are shown in bold.

**Table 2:** Relationship between tertiles of dietary pattern scores and subject characteristics (*n* = 511).

Characteristics	Meat, Fish, and Vegetables Pattern				Milk Pattern						
	Tertile 1 (*n* = 170)	Tertile 2 (*n* = 171)	Tertile 3 (*n* = 170)	*p*- Value ^a^	Tertile 1 (*n* = 170)		Tertile 2 (*n* = 171)		Tertile 3 (*n* = 170)		*p*-Value ^a^
Dietary Pattern Score	−1.12 ± 0.38	−0.08 ± 0.27	1.20 ± 0.86		−1.05 ± 0.56		−0.07 ± 0.20		1.12 ± 0.66		
Age (years)											
70–74	61 (35.9)	71 (41.5)	77 (45.3)	0.512	76 (44.7)		69 (40.4)		64 (37.6)		0.682
75–79	64 (37.6)	61 (35.7)	56 (32.9)		55 (32.4)		64 (37.4)		62 (36.5)		
80–84	45 (26.5)	39 (22.8)	37 (21.8)		39 (22.9)		38 (22.2)		44 (25.9)		
Gender											
Men	42 (24.7)	90 (52.6)	121 (71.2)	**<0.001**	102 (60.0)		68 (39.8)		83 (48.8)		**0.001**
Women	128 (75.3)	81 (47.4)	49 (28.8)		68 (40.0)		103 (60.2)		87 (51.2)		
Education											
Elementary school	110 (64.7)	65 (38.0)	46 (27.1)	**<0.001**	71 (41.8)		95 (55.6)		55 (32.4)		**<0.001**
Higher than elementary school	660 (35.3)	106 (62.0)	124 (72.9)		99 (58.2)		76 (44.4)		115 (67.6)		
Marital status											
Married	98 (57.6)	120 (70.2)	133 (78.2)	**<0.001**	126 (74.1)		114 (66.7)		111 (65.3)		0.168
Others	72 (42.4)	51 (29.8)	37 (21.8)		44 (25.9)		57 (33.3)		59 (34.7)		
Body mass index (Kg/m^2^)	24.4 ± 2.8	24.5 ± 2.7	24.4 ± 3.0	0.856	24.3 ± 2.8		24.3 ± 2.9		24.8 ± 2.7		0.224
Number of physician-diagnosed chronic diseases											
0	38 (22.4)	57 (33.3)	71 (41.8)	**0.004**	61 (35.9)		49 (28.7)		56 (32.9)		0.475
1	81 (47.6)	70 (40.9)	65 (38.2)		64 (37.6)		81 (47.4)		71 (41.8)		
≥2	51 (30.0)	44 (25.7)	34 (20.0)		45 (26.5)		41 (24.0)		43 (25.3)		
Number of prescription drug treatments											
<4	87 (51.2)	94 (55.0)	99 (58.2)	0.425	98 (57.6)		94 (55.0)		88 (51.8)		0.551
≥4	83 (48.8)	77 (45.0)	71 (41.8)		72 (42.4)		77 (45.0)		82 (48.2)		
SGDS-K											
<8	137 (80.6)	154 (90.1)	159 (93.5)	**0.001**	155 (91.2)		145 (84.8)		150 (88.2)		0.191
≥8	33 (19.4)	17 (9.9)	11 (6.5)		15 (8.8)		26 (15.2)		20 (11.8)		
MMSE-KC											
<24	36 (21.2)	27 (15.8)	12 (7.1)	**0.001**	27 (15.9)		28 (16.4)		20 (11.8)		0.419
≥24	134 (78.8)	144 (84.2)	158 (92.9)		143 (84.1)		143 (83.6)		150 (88.2)		
Falls											
Yes	30 (17.6)	32 (18.7)	23 (13.5)	0.398	24 (14.1)		35 (20.5)		26 (15.3)		0.246
No	140 (82.4)	139 (81.3)	147 (86.5)		146 (85.9)		136 (79.5)		144 (84.7)		
Smoking											
Never or former	162 (95.3)	162 (94.7)	159 (93.5)	0.766	160 (94.1)		160 (93.6)		163 (95.9)		0.618
Current	8 (4.7)	9 (5.3)	11 (6.5)		10 (5.9)		11 (6.4)		7 (4.1)		
Dietary supplement use											
Yes	98 (57.6)	102 (59.6)	116 (68.2)	0.102	92 (54.1)		100 (58.5)		124 (72.9)		**0.001**
No	72 (42.4)	69 (40.4)	54 (31.8)		78 (45.9)		71 (41.5)		46 (27.1)		
Energy intake (Kcal/day)	1134.0± 249.2 *	1420.1 ± 265.6 **	1797.7 ± 375.9 ***	**<0.001**	1595.5 ± 428.4 *		1365.3 ± 354.9 **		1391.3 ± 393.9 **		**<0.001**

Values are mean ± standard deviation or number (percentage). ^a^ Analysis of variance (*, **, ***: Results of post-hoc tests) for continuous variables and chi-square test for categorical variables. Abbreviation: SGDS-K-Korean version of the Short Form Geriatric Depression Scale [18]; MMSE-KC-Mini-Mental State Examination in the Korean version of the CERAD Assessment Packet [19]. We marked significant *p*-values in bold.

**Table 3:** Relationship between frailty status and subject characteristics (*n* = 511).

Characteristics	Frailty Status			
	Robust (n = 220)	Pre-Frail (n = 259)	Frail (n = 32)	*p*-Value ^a^
Age (years)				
70–74	117 (53.2)	89 (34.4)	3 (9.4)	**<0.001**
75–79	67 (30.5)	102 (39.4)	12 (37.5)	
80–84	36 (16.4)	68 (26.3)	17 (53.1)	
Gender				
Men	122 (55.5)	122 (47.1)	9 (28.1)	**0.008**
Women	98 (44.5)	137 (52.9)	23 (71.9)	
Education				
Elementary school	79 (35.9)	123 (47.5)	19 (59.4)	**0.006**
Higher than elementary school	141 (64.1)	136 (52.5)	13 (40.6)	
Marital status				
Married	165 (75.0)	168 (64.9)	18 (56.3)	**0.017**
Others	55 (25.0)	91 (35.1)	14 (43.8)	
Body mass index (Kg/m^2^)	24.4 ± 2.7	24.4 ± 2.9	24.8 ± 3.0	0.755
Number of physician-diagnosed chronic diseases				
0	87 (39.5)	73 (28.2)	6 (18.8)	**0.004**
1	91 (41.4)	113 (43.6)	12 (37.5)	
≥2	42 (19.1)	73 (28.2)	14 (43.8)	
Number of prescription drug treatments				
<4	140 (63.6)	133 (51.4)	7 (21.9)	**<0.001**
≥4	80 (36.4)	126 (48.6)	25 (78.1)	
SGDS-K				
<8	210 (95.5)	213 (82.2)	27 (84.4)	**<0.001**
≥8	10 (4.5)	46 (17.8)	5 (15.6)	
MMSE-KC				
<24	18 (8.2)	48 (18.5)	9 (28.1)	**0.001**
≥24	202 (91.8)	211 (81.5)	23 (71.9)	
Falls				
Yes	28 (12.7)	49 (18.9)	8 (25.0)	0.082
No	192 (87.3)	210 (81.1)	24 (75.0)	
Smoking				
Never or former	212 (96.4)	242 (93.4)	29 (90.6)	0.227
Current	8 (3.6)	17 (6.6)	3 (9.4)	
Dietary supplement use				
Yes	135 (61.4)	164 (63.3)	17 (53.1)	0.524
No	85 (38.6)	95 (36.7)	15 (46.9)	
Energy intake (Kcal/day)	1519.3 ± 409.2 *	1421.8 ± 402.1 **	1209.8 ± 287.6 ***	**<0.001**

Values are mean ± standard deviation or number (percentage). ^a^ Analysis of variance (*, **, ***: Results of post-hoc tests) for continuous variables and chi-square test for categorical variables. Abbreviation: SGDS-K-Korean version of the Short Form Geriatric Depression Scale [18]; MMSE-KC-Mini-Mental State Examination in the Korean version of the CERAD Assessment Packet [19]. We marked significant *p*-values in bold.

**Table 4:** Odds ratio (OR) and 95% confidence interval (CI) for frailty and the Fried Frailty Phenotype according to dietary pattern scores ^a^ (*n* = 511).

		Crude OR							Adjusted OR ^b^						
	Tertile 1	Tertile 2		Tertile 3		*p* for Trend	Dietary Pattern Scores (Changes/SD) ^c^	*p*-Value	Tertile 2		Tertile 3		*p* for Trend	Dietary Pattern Scores (Changes/SD) ^c^	*p*-Value
		OR	95% CI	OR	95% CI		OR		OR	95% CI	OR	95% CI		OR	
**Meat, Fish, and Vegetables Pattern**															
Frailty status															
Pre-frail vs. robust	1.00	0.87	0.55–1.37	0.41	0.26–0.65	**<0.001**	0.67	**<0.001**	0.98	0.57–1.67	0.41	0.21–0.81	**0.009**	0.61	**0.004**
Frail vs. robust	1.00	0.57	0.25–1.31	0.14	0.04–0.43	**<0.001**	0.41	**<0.001**	1.25	0.43–3.64	0.38	0.07–1.90	0.289	0.84	0.663
Fried Frailty Phenotype															
Unintentional weight loss	1.00	1.55	0.72–3.32	1.18	0.53–2.64	0.702	0.98	0.902	1.67	0.70–4.00	1.21	0.38–3.89	0.724	0.84	0.534
Exhaustion	1.00	0.59	0.38–0.93	0.32	0.20–0.53	**<0.001**	0.62	**<0.001**	0.77	0.45–1.31	0.41	0.20–0.85	**0.020**	0.62	**0.013**
Low physical activity	1.00	0.93	0.45–1.91	0.56	0.25–1.27	0.173	0.78	0.163	0.95	0.41–2.20	0.61	0.19–1.94	0.426	0.87	0.642
Low grip strength	1.00	1.18	0.74–1.88	0.47	0.28–0.81	**0.008**	0.67	**0.001**	1.59	0.89–2.83	0.79	0.36–1.73	0.667	0.90	0.592
Slow gait speed	1.00	0.74	0.42–1.31	0.45	0.24–0.85	**0.014**	0.68	**0.008**	0.97	0.49–1.95	0.69	0.26–1.80	0.484	0.88	0.594
**Milk Pattern**															
Frailty status															
Pre-frail vs. robust	1.00	1.30	0.84–2.02	0.89	0.58–1.39	0.551	0.89	0.222	1.02	0.63–1.67	0.73	0.45–1.19	0.192	0.83	0.065
Frail vs. robust	1.00	1.15	0.43–3.07	1.52	0.62–3.71	0.337	1.02	0.935	0.51	0.16–1.63	1.17	0.40–3.44	0.572	0.95	0.841
Fried Frailty Phenotype															
Unintentional weight loss	1.00	0.68	0.31–1.47	0.87	0.42–1.81	0.747	0.88	0.421	0.62	0.27–1.40	0.89	0.41–1.95	0.812	0.89	0.492
Exhaustion	1.00	1.35	0.85–2.16	1.26	0.79–2.01	0.379	1.02	0.866	0.96	0.57–1.62	1.07	0.63–1.81	0.772	0.95	0.659
Low physical activity	1.00	1.07	0.51–2.23	0.79	0.36–1.73	0.534	0.95	0.771	0.90	0.41–1.98	0.68	0.29–1.60	0.375	0.91	0.596
Low grip strength	1.00	1.52	0.92–2.51	1.44	0.87–2.39	0.192	1.04	0.672	1.39	0.79–2.43	1.32	0.74–2.33	0.396	0.99	0.906
Slow gait speed	1.00	0.86	0.46–1.59	1.05	0.58–1.90	0.841	1.00	0.998	0.65	0.33–1.30	1.02	0.52–2.00	0.847	1.01	0.975

^a^ Multinomial logistic regression for frailty status, binomial logistic regression for the Fried Frailty Phenotype. ^b^ Adjusted for age, gender, education, marital status, body mass index, number of physician-diagnosed chronic diseases, number of prescription drug treatments, Korean version of the Short Form Geriatric Depression Scale [18], Mini-Mental State Examination in the Korean version of the CERAD Assessment Packet [19], falls, smoking, dietary supplement use, and energy intake. ^c^ Dietary pattern scores/standard deviation. We marked significant *p*-values in bold.

## Supplementary Materials

The following are available online at https://www.mdpi.com/2072-6643/13/2/601/s1, Table S1: Macronutrient intake by tertiles of food group.
