# Supplementary material for: Dietary Patterns and Frailty in Older Korean Adults: Results from the Korean Frailty and Aging Cohort Study
Source: Nutrients. 2021 Feb 12;13(2):601. doi: 10.3390/nu13020601 (PMC7918700; doi:10.3390/nu13020601)
Supplement: Supplementary file 1 [file nutrients-13-00601-s001.pdf]

Table S1. Macronutrient intake by tertiles of food group (N=511).

Variable	Seasonings			
	**Tertile 1**	**Tertile 2**	**Tertile 3**	***p*-Value**[a]
Percent of energy[b]				
From carbohydrate	68.9 ± 8.7	66.5 ± 7.8	65.5 ± 7.1	<0.001
From protein	14.4 ± 2.7	15.4 ± 3.0	16.1 ± 2.8	<0.001
From fat	16.7 ± 7.1	18.1 ± 6.1	18.3 ± 5.9	0.031
Energy (Kcal/day)	1246.0 ± 323.9	1425.4 ± 331.2	1683.6 ± 408.5	<0.001

Values are mean ± standard deviation. [a]Analysis of variance for continuous variables. [b]Adequate energy ratio from carbohydrate 55-65%, protein 7-20%, and fat 15-30% in 19 years old or older [26].
